# Supplementary material for: Are carbon emissions trading and green financial instruments synergistic? -Comprehensive quantitative research based on content analysis
Source: PLoS One. 2024 Mar 7;19(3):e0298601. doi: 10.1371/journal.pone.0298601 (PMC10919601; doi:10.1371/journal.pone.0298601)
Supplement: S1 Data — (ZIP) [file pone.0298601.s001.zip › Data/地级市Z_F工作报告.zip!/║╙▒▒/└╚╖╗╩╨/└┤╫╘╣1⁄2╓┌║┼í╛┬φ┐╦╩2╛▌═°í┐_2017.docx]

过去四年 主要工作总结

4月11日，市长陈平在市七届人大一次会议上作政府工作报告时说，2013年市六届人大一次会议以来，在省委、省政府和市委坚强领导下，在市人大、市政协监督支持下，市政府坚持以习近平总书记系列重要讲话精神为指引，主动适应经济发展新常态，深入贯彻发展新理念，全力抢抓京津冀协同发展重大机遇，积极推进供给侧结构性改革，与全市人民一道，脚踏实地，真抓实干，较好完成各项目标任务，为全面建成小康社会奠定了坚实基础。

(一)牢固坚持稳中求进总基调，主要经济指标进一步向好。四年来，地区生产总值由1794.3亿元增加到2706.3亿元，年均增长8.5%，增速居全省第1位；全部财政收入由306.6亿元增加到595.5亿元，年均增长18.1%，增速居全省第1位；一般公共预算收入由172.1亿元增加到338.7亿元，年均增长18.4%，增速居全省第2位；全社会固定资产投资由1314.1亿元增加到2490.8亿元，年均增长18.2%，增速居全省第1位；规模以上工业增加值由673亿元增加到776.5亿元，年均增长7.5%，增速居全省第3位；存款余额由2714.4亿元增加到6179.9亿元，年均增长31.6%，贷款余额由1875.3亿元增加到4905.2亿元，年均增长27.2%，增量、增速均居全省第1位。特别是全部财政收入总量，由全省第6位跃居第3位；一般公共预算收入总量，由第4位升至第3位。全部财政收入占GDP比重达到22%，一般公共预算收入占GDP比重达到12.5%，税收占GDP比重达到19.7%，均居全省第1位。

(二)加快产业转型升级，产业结构进一步优化。四年来，三次产业结构由11:54:35调整到7.3:44.1:48.6，实现了从“二三一”到“三二一”的历史性转变。战略性新兴产业加快发展。累计实施亿元以上战略性新兴产业项目642个，完成投资1914亿元，分别占亿元以上产业项目的31%和28.7%；实施的省重点项目中战略性新兴产业项目数量占到85%、投资额占到68.9%。润泽信息港、华为云计算、华夏AMOLED、京东电子商务产业集群、联通华北基地、新奥泛能网等“大智移云”标志性产业项目聚集发展；香河机器人产业港、泉恩管材等高端装备制造业规模发展；中安信碳纤维、康得复合材料、碳纤维宝马车体等新材料产业壮大发展；科达装备、航天振邦等军民融合产业加快发展。服务业增势强劲。服务业增加值由627.8亿元增加到1315.3亿元，对经济增长的贡献率由32.4%提高到70.4%。现代服务业发展加快，大厂影视创意产业园、微软游戏创新中心等文化产业项目建成投运，燕达国际健康城、爱晚养老基地等健康养老产业项目初具规模，鲁能生态区等旅游业项目加快建设。金融服务业不断壮大，2016年实现增加值229.4亿元，占服务业增加值比重达到17.4%。传统产业改造升级步伐加快。累计实施技改项目2585项，技改投资达到2495.1亿元，年均增长20.1%，居全省第1位。河北钢木家具技术研究院、河北绝热保温建材产业研究院等省级产业研发平台建成投用，企业创新能力增强，传统产业的信息化、智能化水平逐步提升。现代农业发展水平不断提高。省级以上农业科技园区、现代农业园区达到19家，省级以上农业产业化龙头企业达到49家。全市域被纳入京津冀都市现代农业区，6个县(市、区)被列入环首都现代农业科技示范带。

(三)加快对接京津，协同发展进一步推进。交通、产业、生态三大重点领域协同实现突破。津保铁路、京台高速、密涿高速(廊坊段)建成通车，京廊通勤高铁开通运行；平谷线(三河段)开工奠基，京霸、京唐、廊涿固保、城际铁路联络线四条轨道交通项目前期工作基本完成。京东华北订单中心、京东方二期等产业转移项目建成投产，中核研发中心、中粮可口可乐等重点项目落地建设，四年累计引进京津项目830个、资金1590亿元。与京津实施大气污染防治联防联控，和大兴、武清建立区域联动水污染防治机制，“平三蓟”京津冀国家生态文明先行示范区获国家批复。重要承接平台加快布局。顺利完成北京新机场土地征迁、河流改道等相关工作，确保了国家重点工程按时开工建设。主动配合国家和省推进临空经济区规划建设。积极配合北京编制通州与北三县整合规划。北京亦庄·永清高新区、京冀通航产业园等合作共建园区顺利推进，“通武廊”战略合作框架协议正式签订。协同创新共同体加快建设。按照“北京研发、廊坊转化”思路，建立了清华大学中试孵化基地、北京大学科技成果展示交易中心等15个科技成果孵化转化基地和全国第一家博士后成果转化基地。制定出台科技创新“1+4+N”系列文件。全市新认定科技型中小企业4303家、科技小巨人企业276家、高新技术企业192家；省级以上高新区、孵化器分别达到6家和11家，均居全省第1位。科技进步贡献率达到48%，我市被评为全国科技进步先进市。

(四)加大污染防治力度，环境质量进一步改善。打响蓝天保卫战。坚持专家引路科学治霾、每周集中调度督导、春夏秋冬分类施策、污染天气超前应对、市县乡村四级联防联控。深入开展“蓝天行动”“利剑斩污”“月清百污”等系列专项整治，强力减煤、抑尘、限车、治企、控烟。完成25.9万户“散煤替代”工程设备安装，淘汰市县建成区5110台10蒸吨及以下燃煤锅炉，取缔126座实心粘土砖瓦窑，取缔关停1700余家非法排污企业，完成183家涉VOCs企业治理。强化机动车管控，主城区实施公交车免费，淘汰15.9万辆黄标车。空气质量综合指数在74个国控城市中倒排第12位，成功退出“倒排前十”。全域实施生态修复。四年累计造林167.7万亩，森林覆盖率达到28.91%，提高14.2个百分点。三河矿山修复全面展开，香河潮白河大运河湿地公园列入国家试点。强力治理水环境污染。国家环保部于2016年10月正式解除我市涉水项目限批。

(五)深入推进改革开放，发展活力进一步增强。纵深推进“放管服”改革。全部取消130项市本级非行政许可审批事项，衔接取消下放行政审批事项316项，“多证合一、一照一码”全面实施，电子营业执照全行业试行，工商前置许可审批事项减少82.9%，市场主体由14.4万户增加到26.9万户。稳步实施财税金融改革。“营改增”全面推开，绩效预算制度逐步健全，全市挂牌上市企业由8家增加到65家。扎实推进医药卫生体制改革。全市8个县(市)、19家二级医院纳入公立医院改革试点，实行药品零差率销售。强力推动开发区改革。省级以上开发区由32个优化整合为14个；廊坊开发区体制机制改革经验在全省推广；固安工业园PPP模式获国务院办公厅通报表扬。成功争列第一批全国中小城市综合改革试点，全面完成市县政府机构改革、党政机关公车改革，农业农村、供销社、国企、教育、文化等领域改革迈出新步伐。着力提高开放水平。累计实际利用外资29.6亿美元，63家企业实现“走出去”；连续成功举办“5·18”经洽会、“9·26”农交会等国家级展会，被评为中国十佳优秀会展城市。

(六)加快推进新型城镇化建设，城乡面貌进一步改善。全市常住人口城镇化率达到56.8%，居全省第3位。中心城市功能日趋完善。高铁站南广场、金光道地下通道等基础设施投入使用，浙商广场、香港新世界等商业设施建成运营，建设提升一批城市道路和公共服务设施。国电热电联产一期提前投入运行，新增供热面积900万平方米，对去年大气环境质量有效改善举足轻重。中电投燃气热电联产项目正式获批。垃圾发电厂建成投用。在全省率先实施市县两级数字城管，主城区重点场所热点区域免费WiFi全覆盖，我市被列为国家智慧城市试点。县域发展水平快速提升。所有县(市、区)全部财政收入均迈上10亿元台阶，6个县(市)进入全省民营经济十强。开展县城和重点镇提升行动，城镇功能不断完善。美丽乡村建设扎实推进。投资68.5亿元，连续实施“十二项专项行动”，建设了北三县和固安、霸州三大省级重点片区，完成了1116个村街改造提升，全省美丽乡村建设现场会连续两年在我市召开。

(七)着力改善民生，人民群众获得感进一步提升。四年财政民生支出累计达到1284.3亿元，占一般公共预算支出的81%。民生保障得到加强。城镇累计新增就业岗位23.5万人，城镇登记失业率连续保持全省最低，城乡居民人均可支配收入分别达到34633元和14286元，居全省第1和第2位。城乡居民基本医疗保险整合全面完成；城乡低保标准分别达到每人每月532元和354元，居全省第2和第1位。在全省率先实施重特大疾病救助制度，累计支出救助基金1.13亿元。开工建设保障性住房5.2万套，改善老旧小区108个，改造农村危房1.2万户。建设农村集中供水水厂138座，在全省率先解决农村饮水安全问题。社会事业全面进步。新改扩建主城区中小学和幼儿园16所、农村幼儿园107所，改善义务教育薄弱学校504所，标准化学校创建率达到75%。燕达医院与北京4家医院实现共建共管，率先建立医保异地就医结算机制；实施健康京畿·中医药先行“8·10”工程。我市被授予全国公共文化服务体系示范区。社会大局和谐稳定。持续加大安全生产、食药安全工作力度，有效防范重大安全事故；扎实推进法治廊坊、平安廊坊建设，社会治安综合治理能力不断提升，在全省率先建成四级群众工作中心(站、室)，信访形势趋稳向好；圆满完成APEC、纪念抗战胜利70周年大阅兵等重大活动安保任务。

今后五年工作总体要求和主要目标任务

关于今后五年工作总体要求和主要目标任务，陈平说，市第六次党代会明确提出了今后五年全市工作的指导思想：深入贯彻党的十八大和十八届三中、四中、五中、六中全会精神，坚持以习近平总书记系列重要讲话精神引领航向，全面落实省第九次党代会决策部署，认真践行创新、协调、绿色、开放、共享的发展理念，积极适应和引领经济发展新常态，大力推进供给侧结构性改革，抢抓新机遇，率先走新路，着力补短板，强力促创新，做强大平台，继续解放思想、奋发进取，坚持干字为先、狠抓落实，打造科技研发创新成果转化引领区、战略性新兴产业和现代服务业聚集区、京津冀全面创新改革试验区、北京非首都功能重要承载地，奋力推动经济发展、转型升级、生态建设、民生保障、维护稳定走在全省前列，为提前全面建成小康社会、加快建设创新驱动经济强市而奋斗。市第六次党代会为做好政府工作指明了方向，我们要坚决按照市委的部署要求，狠抓推进落实，确保如期完成。

未来五年，主要战略任务是提前全面建成小康社会和加快建设创新驱动经济强市。2016年底，全市市级监测的全面建成小康社会31项指标中，已达到或超过标准的有18项，还有人均GDP、环境质量指数、卫生发展指数、人均公共文化财政支出等13项指标未达到标准。加快建设创新驱动经济强市，还必须在科技进步贡献率、研发机构数量、万人发明专利拥有量、规模以上高新技术产业增加值占规模以上工业增加值比重等指标上实现大幅提升。

未来五年，主要经济社会发展目标是全面落实市第六次党代会要求：地区生产总值年均增长8%以上，人均生产总值力争达到1.4万美元，位居全省前列；一般公共预算收入年均增长8%，达到550亿元；固定资产投资年均增长15%左右。规模以上高新技术产业增加值占规模以上工业增加值的比重达到35%以上。战略性新兴产业对经济增长的贡献率超过传统产业；“大智移云”产业年均增长20%以上，产业规模突破3000亿元，保持全省首位；服务业增加值占GDP比重达到58%左右，位居全省前列。空气质量稳定退出全国“倒排前十”，PM2.5平均浓度降至55微克/立方米以下，在京津冀13个国控城市中下降率位居前列，全市优良天数比例达到72%以上，实现空气质量持续改善；水环境治理达到国家和省考核要求的较好水平，全市森林覆盖率稳定在30%以上。城乡居民人均可支配收入年均增长8%以上，总量保持全省领先，增速快于经济增长，多数县(市、区)收入水平看齐京津。城乡居民社会保障水平全省领先，城镇登记失业率保持全省最低，城乡低保标准保持全省最高，教育、医疗等基本公共服务水平大幅提升，与京津差距明显缩小。新型城镇化建设全面提速，城乡面貌、承载能力和公共服务均等化水平显著提升，美丽乡村建设在全省率先实现全覆盖，全市常住人口城镇化率达到62%以上。公共安全和社会治安综合防控体系及群众工作体系更加健全，信访工作在全省领先，安全生产、食品药品安全各项控制指标保持全省先进水平。

做好政府今后五年的工作，最根本的是始终坚持与以习近平同志为核心的党中央保持高度一致，坚定不移以习近平总书记系列重要讲话精神和对河北的指示要求引领航向，坚持稳中求进工作总基调，用新发展理念指导实践推进工作，以实际行动体现对党绝对忠诚。最要紧的是始终坚持以人民为中心的根本立场，坚定不移走群众路线，把人民群众满意不满意、高兴不高兴、答应不答应作为想问题、做决策、办事情的出发点和落脚点，真正做到权为民所用、情为民所系、利为民所谋，以实际效果证明人民政府就是为人民服务的。最基础的是始终坚持把实现协同发展作为战略基点，坚定不移贯穿到创新发展、协调发展、绿色发展、开放发展、共享发展的各个领域，在对接京津、服务京津中加快发展自己，以实际成果加快与京津一体化、同城化步伐。最迫切的是始终坚持落实省委、省政府对廊坊发展做出的新要求新决策毫不含糊，坚定不移发挥自身优势，抢抓历史机遇，在全省率先走出“加快转型、绿色发展、跨越提升”的新路，以实际成效让省委省政府放心、让全市人民满意。最关键的是始终坚持市委的坚强领导，坚定不移贯彻市第六次党代会精神，完成好市委提出的各项目标任务，以实际成就凸显廊坊发展走在全省前列。最实际的是始终坚持事业是实干出来的工作理念，坚定不移践行实字当头、干字为先，定了就干、干就干好、干就干成，以实打实、硬碰硬的作风展示新一届政府新形象。

2017年目标任务和重点工作

关于2017年目标任务和重点工作，陈平说，五年看头年，开局最关键。今年是实施“十三五”规划的重要一年，是供给侧结构性改革的深化之年，也是全面落实省市党代会精神的开局之年，特别是将迎来党的十九大胜利召开。做好今年工作，完成今年任务，责任重大、意义深远。

今年全市经济社会发展的主要目标是：地区生产总值增长8%以上，一般公共预算收入增长8.5%左右，固定资产投资增长15%左右，服务业增加值增长13%左右，规模以上高新技术产业增加值增长15%左右，社会消费品零售总额增长10%以上，实际利用外资增长4.7%，城乡居民人均可支配收入均增长8%左右，单位GDP能耗、主要污染物减排、空气质量改善绩效指标完成省下达目标任务。

实现今年目标任务，必须以强烈的历史责任感，抢抓京津冀协同发展一系列交汇叠加的重大机遇，必须以新发展理念引领经济社会发展全局，坚决做好以下方面工作：

(一)更加注重协调，着力深入推进和融入协同发展。

一是强化与京津区域规划对接协调。加强全域规划精准衔接。立足国家和省协同发展规划中对我市的基本定位，支持北三县主动服务和融入北京城市副中心建设，积极配合北京做好《通州及廊坊北三县地区整合规划》，抓紧调整提升功能定位，严格控制房地产业发展规模和开发强度，在环境治理、设施配套、互联互通等方面和通州做好主动对接、统一标准，努力打造文创与高端服务业大区；支持主城区和永清、固安依托北京新机场和临空经济区建设，加快组团发展，强化与临空经济区总体规划及各专项规划的对接，做大做强城市经济和临空产业，打造科技成果转移转化与高端临空产业大区；支持南三县积极对标、主动服务雄安新区，对现有城镇、产业、生态等各项规划，进行重新审视、重新定位、重新再造，构建和新区规划衔接、功能匹配、协同发展新格局，在对接和服务中加快打造全市新的经济增长极。严格执行各项管控措施。坚决服务服从协同发展大局，全力抓好京廊交界地区和雄安新区周边管控，对相关区域内的产业发展、城镇建设、生态环保进行清单管理，一定避免房地产“围城式”发展，严厉打击房地产市场恶意炒作、虚假广告宣传、黑中介违法违规等行为，妥善处置问题楼盘，确保房地产市场平稳健康发展。

二是强化与京津交通基础设施对接协调。积极做好京霸、京唐、廊涿固保、城际铁路联络线等轨道交通项目开工前的相关工作，做好平谷线(三河段)建设配合工作，启动环北京城际廊坊至香河段、霸州至衡水段建设前期工作，着力建设“轨道上的京津廊”。积极推进北京新机场配套交通路网建设，力促机场北线高速、105国道京冀界至永清段年内开工，做好机场南出口高速前期工作。配合推进京秦高速京津连接线建设和津石、唐廊高速前期工作，确保密涿高速万庄连接线建成通车。实现潮白河大桥竣工通车。

三是强化与京津城市基础功能对接协调。着眼建设京津冀世界级城市群重要节点城市，对标承接非首都功能疏解和产业转移，加快提升城镇建设管理水平，促进城镇建设与京津同城化一体化发展。做大做强中心城市。开展主城区改造提升三年行动，进一步拉大城市框架，完善基础功能，提升形象品位，增强承载力和竞争力。实施棚户区改造工程。对125个城中村和危陋住宅区全面征迁改造，年内完成28个城中村和11个危陋住宅区近2万户的征迁。实施交通疏堵保畅工程。要全力贯通“大动脉”，加快启动外环路西、南环线开工建设，年内实现大外环彻底合拢；提升第三南通道建设水平，打通主城区到胜芳高铁站的快捷线；全线贯通艺术大道并西延至西环路，使艺术大道真正成为“大道艺术”。要加力打通“中梗阻”，彻底打通总长54.7公里的17条沉睡已久的“断头路”，年内要高标准完成11个段区、18.5公里。要努力畅通“微循环”，年内全面实施胜利街等99条小街巷改造。实施功能提升工程。加快朝阳公园、瑞丰公园建设，填补市区东部、南部没有综合性公园的空白。鼓励使用电动新能源汽车，完善配置充电网络，新建快充站22座。大力解决停车难问题，新建立体停车场12个，开放机关事业单位停车场43个，整合社会机构停车场76个，新增停车位16300个。加快海绵城市建设，完成20平方公里海绵城市试点和10公里综合管廊建设。加快完成市殡仪馆迁建。实施精细化管理工程。提升城市绿净亮美水平，投资18.6亿元，实施一批道路绿化、重要节点绿化、公园游园建设提升项目，推行立体绿化，打造花海景观，增加城市绿量；按照城市主干道标准提标改造廊涿引线，建成标志性精品景观大道；打造广阳道、北凤道、新华路、银河路4条精品夜景示范街，营造190个单体建筑夜景，扮靓动静相宜的城市夜空；优化城市环卫管理，将环卫清扫职能下放广阳、安次两区。强化智能化管理，建立城市管理综合指挥中心，对城市管理实施全方位动态监控、调度、指挥，建立全覆盖的城管指挥联动体系。加大综合执法力度，整合住房、城建领域和环保、工商、交通、水务、食药监部分行政处罚权集中行使，实现一支队伍管执法；强力拆除违章建筑，重点整治占道停车、店外经营、乱贴乱画等行为。着力打造城市特色，开展城市符号设计，规范城市建筑风格，对城市色彩、建筑、街道、绿化、照明等进行规划控制，塑造主城区整体形象，彰显城市文化内涵和魅力特质。推进县城建设上档升级。围绕提升综合承载能力，坚持“小县大县城”战略，实施县城建设攻坚，加快完善基础设施，增强公共服务能力，推进县城建设与京津功能互补、联动发展。完善城乡规划体系，优化县域空间布局，积极推进“多规合一”。加强基础设施建设，启动实施160项重点城建项目。提升县城环境容貌，开展“洁净城市”创建，综合整治县城环境，每个县新建或改造提升1个以上特色风貌街区、2个以上精品建筑，提升县城品质品位。调整优化功能定位，支持三河、大厂、香河、固安、永清建设特色鲜明、设施完善、生态宜居的环首都卫星城；支持三河、霸州打造50万人以上的中等城市，建设京津冀城市群特色节点城市；支持霸州、文安、大城构建与白洋淀水脉相接、森林一体的区域生态体系，打造生态环境优美、文化底蕴深厚、产业支撑有力的新增长极。加快特色小镇建设。重点推进香河运河文化创客小镇、安次北田曼城国际小镇、广阳西部金融小镇等10个省级特色小镇建设。

四是强化与京津收入分配、基本公共服务政策对接协调。搞好调查研究。重点针对个人收入、义务教育、公共卫生和基本医疗、社会保障、公共就业服务等群众最关心最直接的现实问题，摸清底、对好标、算好账。坚持整体推进重点突破。整体推进就是统筹兼顾考虑到面，保持公共服务的普惠性和平衡性；重点突破就是实力强的县先行，条件成熟的县快行，率先试点提供借鉴，不搞一刀切、简单化。以国家级改革试验区为抓手，鼓励尝试“一市两制”“一县两制”，直接对标看齐京津，在引资引智、要素供给、管理服务等相关政策方面实现与京津均等化、均质化，加快区域市场要素一体化进程。

五是强化与京津合作共建项目对接协调。以临空经济区建设、北三县统筹发展两大历史性工程，以及北京亦庄·永清高新区等合作共建平台为重点，进一步健全与京津的沟通对接机制，不断完善和加强定期互访、重点事项会商、互派干部交流等工作制度，努力形成政府间常态沟通、对口部门紧密衔接、专项工作协同推进的良好态势，加快推动合作共建重大项目取得实质性进展，为承接非首都功能和产业转移疏解提供载体和平台。

(二)更加崇尚创新，着力加快产业转型跨越提升。

一是大力增强产业创新“动能”。加大去产能力度。钢铁去产能是严肃的政治任务，必须坚决去、主动调、加快转，9月底前确保成建制退出1家，力争年底前再退出1家。同时，全面对接落实协同发展规划和生态环保要求，提升行业和区域准入门槛，运用市场化法治化手段，倒逼落后低端产业加快退出市场，严格限制承接和布局一般性制造业。持续壮大战略性新兴产业。大力发展“大智移云”产业，加强润泽信息港、华为云计算等已有大数据产业的开发应用，确保京津冀大数据应用感知体验中心“5·18”前建成投用，打造全国领先的大数据应用先行区。力促华夏AMOLED项目完成主体工程，京东电子商务产业集群5个项目加快建设，菜鸟智能骨干物流、中鼎云等重点项目早日开工，推动“大智移云”产业加快集聚规模发展。大力发展新材料和环保产业，推动中安信碳纤维二期、康得复合材料、恒盛水处理设备等项目建成投产，提高新型纤维材料、新型纳米材料、节能环保等产业的核心竞争力。大力发展航天航空产业，力促“河北一号”小卫星群项目尽快开工，推动郎创航天项目加快建设，全力争取中科院遥感所在我市设立产业研究院，支持航天军民融合成果转化基地提高孵化转化能力。大力发展高端装备制造、新能源等战略性新兴产业。加快发展现代服务业。推动金融业发展，积极争取京津新型金融业态在我市设立分支机构，壮大融资租赁、资产管理、股权投资基金等新兴业态。推动文化产业发展，依托大厂影视创意产业园、大城红木文化新区等重点项目，做大做强动漫游戏、影视制作等文创产业。大力发展会展经济，加快国际会展中心改造提升和会展新区谋划建设，积极对接北京会展资源，引进举办一批市场化、专业化、国际化的品牌展会，全年举办各类展会40个以上。推动健康养老产业发展，立足服务京津养老需求，重点发展健康养老与医疗服务相结合的中高端健康养老产业，支持燕达国际健康城、爱晚养老基地等龙头企业发展壮大。推动智慧物流、科技服务、信息服务等现代服务业加快发展。升级改造传统优势产业。实施技改项目500项，新增1-2家省级以上新型工业化产业示范基地。加快“互联网+传统产业”改造提升步伐，完善“企业云”平台功能，提升传统产业智能化、信息化水平。深入实施质量强市战略，开展增品种、提品质、创品牌“三品”行动，积极创建全国质量强市示范城市。不断提升农业发展质量效益。推动一二三产深度融合，大力发展高端设施农业、生态高效农业、休闲观光农业和农产品加工物流业，新增市级农业产业化龙头企业40家。依托环首都现代农业科技示范带建设，力争共建京廊农业科技协同创新平台和成果转化基地9个，培育农业科技小巨人企业40家。

二是大力增强产业创新“势能”。加快提升科技创新能力。实施人才兴廊工程，积极引进高端人才和创新团队，新建院士工作站3家，引进培育高层次创新团队12个以上。加强创新平台建设，新增孵化器和众创空间20家以上、孵化面积20万平方米，新建市级以上研发中心100家、技术创新战略联盟10家。加大创新投入力度，全市规模以上工业企业研发投入增长20%。加快提升企业创新能力。大力培育创新型企业，继续实施苗圃、小壮大、小升高、企业上市四大工程，新增科技型中小企业1300家、科技小巨人企业85家、高新技术企业60家、挂牌上市企业25家。鼓励企业开展技术创新，支持华为、中兴、京东方等行业领军企业进行关键技术研发，努力打造能够在廊坊落地生根的产业品牌；支持新奥、华夏、荣盛、宏泰等本土优势企业并购、引进域外高新技术企业和研发机构，开发一批战略产品，形成一批创新型产业集群。加快提升协同创新能力。依托京南国家科技成果转移转化示范区、京津冀大数据综合试验区，加快清华大学中试孵化基地、廊坊·中关村软件园人才与产业创新基地等协同创新基地建设，新建各类协同创新基地20家；加强与京津高校院所和创新团队的对接合作，全年转化北京先进科技成果50项以上。

三是大力增强产业创新“效能”。深入落实“1+4+N”系列文件，进一步释放“双创”活力。加强知识产权保护，完善科技成果市场定价、收益分配、转移转化评价机制，进一步增强创新动力。持续增加财政科技投入，扩大科技基金规模，市级设立2-3支不低于20亿元的子基金，每个县(市、区)建立不低于1亿元的创业创新投资基金，为科技企业和创新人才提供低成本、便利化的融资服务。

(三)更加倡导绿色，着力加强环境保护和污染治理。

一是打好大气污染防治治本攻坚战。严格落实省“1+18”大气污染防治系列文件要求，坚持和强化依法治霾、协同治霾、科学治霾、铁腕治霾，把优化产业结构、能源结构、交通结构作为重点，推动大气污染治理由治标为主向治本为主转变，确保PM2.5浓度降到62微克/立方米左右，稳定退出全国“倒排前十”。强力推进散煤综合治理。继续实施“煤替代”工程，确保9月底前完成荣乌高速以北禁煤区农村地区40余万户的替代任务；同时，力争完成荣乌高速以南霸州、文安、大城农村地区30余万户的替代任务，实现全市域散煤“清零”，并严格取缔散煤销售网点。加强燃煤锅炉治理，9月底前实现全市域10蒸吨及以下和市建成区35蒸吨及以下燃煤锅炉“清零”；扩大城市主热网覆盖面，年内新增集中供热面积300万平方米以上；高标准改造提升供热站点，加快启动中电投燃气热电联产项目建设，确保2018年主城区全部实现清洁集中供热。强力推进工业污染治理。坚持工程减排、结构减排、管理减排多措并举，对重点行业实施深度治理和错峰生产，加强污染物传输通道高架源企业治理。坚持改造提升和退城并重，有序推进园区、县城建成区污染企业退出。开展工业污染源全面达标排放行动，实施大气污染特别排放限值，所有排放企业强制统一安装在线监测系统，重点污染源实现稳定达标排放。强力推进车辆油品治理。大力发展公共交通，在市区广阳道、和平路、银河路设置公交专用道，新增新能源公交车100部，采取公交免费措施，鼓励市民多乘公交车、少开私家车；所有加油站(点)9月底前全部供应符合国六标准的车用燃油；科学实施外埠大型货车远端分流和近程管控。强力推进烟尘综合治理。开展扬尘综合整治百日专项行动，全面落实建筑工地扬尘防治“七个100%”，道路机械化清扫率达到85%以上，市区实现“以克论净”全覆盖；规模以上土石方建筑工地开工前必须实现在线监测，城市建成区全面禁止露天烧烤；严格落实烟花爆竹禁限放、垃圾秸秆禁烧等措施；倡导移风易俗、文明祭祀，主城区禁烧祭祀用品。

二是打好生态修复持久战。加大水污染治理力度。深入贯彻国家“水10条”和省“水50条”，全面落实市县乡河长制，加强6条国省控和13条市控河流综合治理。强力开展主城区环城水系、城市黑臭水体综合整治，真正让环城水系活起来、动起来、美起来。加快主城区污水处理设施建设，以市建设局和广阳区、安次区政府为责任主体，年内力争高标准建成铁路北、万庄、凯发新泉二期三座污水处理厂，新增日处理能力15万吨。全面取缔涉水“十小”企业，强力开展十大重点行业用水清洁化改造。以各县(市、区)政府为责任主体，年内完成省级以上园区19个污水处理厂建设，实现省级以上园区污水处理厂全覆盖。加大水资源优化配置力度。加快南水北调水源切换，已引入长江水的县(市)要确保稳定运行，未完成水源切换的确保4月底前完成切换并通过省验收；市建设局要积极做好主城区水源切换试水工作，确保6月底前主城区全部用上长江水。加快实质推进南水北调调蓄工程广阳水库建设，改变全省唯独廊坊没有地表水库的局面。加快推进燕郊应急调水相关工作。加大土壤污染治理力度。开展土壤污染调查、风险评估和治理修复，实施危险废物治理、涉重金属企业清理整顿等专项整治；加强农业面源污染防治，实施化肥、农药零增长行动，保障土壤环境安全。加大造林绿化力度。积极推进环首都生态保护绿带建设，高标准实施天堂河“一河两岸”绿廊工程，全市植树造林25万亩，争创国家森林城市。加快香河潮白河大运河国家湿地公园、文安赵王新河省级湿地公园建设，恢复、改造提升湿地1.3万亩。运用产业化方法抓生态建设，依托湿地资源加快推进鲁能生态区等项目建设，打造特色休闲旅游功能区。

三是打好“法治生态”速胜战。加强与京津在生态环境立法、司法领域合作，持续开展跨区域环境保护联动执法。严格落实新环保法及省大气污染防治条例等法律法规，保持环境违法严打高压态势，依法治理“土小”产业群和“散乱污”企业，6月底前取缔关停4600家以上“散乱污”企业；全面实施重点行业VOCs达标治理，建立重点行业排放清单信息库，6月底前全面完成对标管理和深度治理。实施污染治理风险抵押金制度。加快生态环境立法，对环境准入、环保职责等进行明确界定。

(四)更加厚植开放，着力优化市场营商环境。

一是不断优化行政服务。进一步深化“放管服”改革。及时衔接国家和省取消下放行政审批事项，清理规范行政审批中介服务事项，最大程度下放市本级行政权力事项。完善清单管理制度，编制公开市级行政许可事项目录清单，推动行政权力依法公开规范运行。加快覆盖全市的便民服务体系建设，积极推进“互联网+政务服务”，开展网上行政审批试点。持续推进商事制度改革。积极实行“六证合一”，探索实施“证照联办”，推进工商注册便利化，开展个体工商户简易注销试点；加强事中事后监管，实现“双随机、一公开”监管方式全覆盖。全年新增市场主体4.8万家。加快组建行政审批局。4月底前，市县两级全部挂牌试运行。

二是扎实做好重点领域改革。加快省级以上改革试点建设，重点推进石保廊全面创新改革试验，力争形成1-2项在全省可复制、可推广的经验；深入推进国家中小城市综合改革试点，力争园区管理运营模式、投融资平台建设等工作在全省、全国形成典型经验。深化财税体制改革，推进地方财政事权与支出责任划分，完善预算科学管理；创新融资平台，成立市级政府投资基金，引导社会资本共同组建“子基金”，放大财政资金“乘数”效应；推动县级信用联社改制农村商业银行；建立重大项目融资咨询机制，搭建项目建设融资服务平台。强化经营性国有资产集中统一监管。加强信用联动奖惩，构建新型市场监管机制。尽快推开市级机关内设机构改革，推进事业单位和国有企业公车改革，加快农业农村、供销社、科教文卫等领域改革。

三是提升对外开放水平。做大做强开发区平台。充分发挥廊坊经开区的示范引领作用，支持通过托管和扩区方式拓展发展空间，放大国家级开发区品牌效应。落实国家和省促进开发区改革的政策措施，推广廊坊经开区改革经验，所有开发区4月底前完成人事薪酬制度改革、投融资平台建设等改革任务；加强开发区配套建设，加快由水、电、气等“九通一平”向信息通、市场通、法规通等“新九通一平”提升。力争廊坊出口加工区升级综合保税区，新陆港铁路口岸列入省陆路口岸发展规划。大力开展招商引资活动。以各县(市、区)、开发区为主体，坚持招商引资与招才引智并举，把京津作为主攻方向，引进符合我市产业定位的新项目、大项目和创新要素，完善重点产业链条、培育新的产业集群，今年实施亿元以上项目不低于1000个。全力以赴办好“5·18”经洽会，高水准举办专题论坛、成果展示、项目签约等重点活动，努力在组织形式和招商实效上实现革命性变化。继续办好“9·26”农交会等重大会展活动，争取军民融合展洽会升级为国家级展会。加快推动优势富余产能“走出去”。鼓励企业以对外投资、承包工程为重点，积极开展国际产能合作，帮助钢铁企业省外国外建厂；支持大型民营企业建设境外产业新城、产业园区，带动关联企业“抱团出海”。

四是着力推动民营经济加快发展。积极为品牌企业拓宽投资领域。深入落实国家、省促进民间投资的各项政策，鼓励民营资本以股权合作、PPP等方式，积极参与铁路公路交通、污水垃圾处理、医疗教育卫生等城乡重大基础设施和社会公用事业建设。支持企业参与重点园区开发，加快建成一批一二三产有机融合的新型经济业态和产城教融合发展的现代产业示范园区。积极为成长性好的中小型企业加油助力。围绕改造提升县域传统优势产业集群，鼓励地方龙头企业对当地同类企业进行整合提升再造，或引进战略投资者以多种形式对本土企业进行战略重组，带动相关企业不断提升经营管理水平，完善法人治理结构，建立现代企业制度，积极上市融资，培育壮大一批集团化、股份合作化、管理现代化的行业领军企业集团，带动产业链上下游企业提升专业分工、联合协作水平，推动食品、家具等优势传统产业上档升级、转型发展。加强民营企业家队伍建设。实施中小企业家素质提升工程。

五是打造先进的营商文化。先进的营商文化，能够塑造优良的企业精神，营造优质的商业环境，传承优秀的商道精神，激发民营经济发展的内生活力。营造遵纪守法、“亲”“清”新型的政商文化。加快法治政府和服务型政府、法治社会和诚信社会建设，构建政府与企业良性互动的政商机制和环境，坚决消除各种“潜规则”，坚决拒绝各种“黑交易”，努力建设“亲”“清”新型政商关系。营造情系家乡、回报社会的企业家文化。强化企业家富而思源、建设家乡的责任担当，鼓励企业家投资家乡，带动老乡共同富裕，为家乡建设增砖添瓦；培树企业家富而思善、回报社会的道义情怀，支持企业家多办慈善事业、多做爱心工程，为社会传递正能量。营造精诚团结、加强协作的竞合文化。要积极倡导企业家互相尊重、互相学习，沟通合作、抱团发展，坚决避免互相贬低、恶性竞争、“使绊子”“窝里斗”，努力在竞合中共面难题、共享机遇、共同发展。营造包容亲和、海纳百川的亲商文化。对外来投资者，既要坚守公道公平公正的营商之道，又要胸怀“有朋自远方来不亦乐乎”的宽宏气度，坚决克服“自耕一亩三分地”“肥水不流外人田”的狭隘地域观念。政府要引领社会，对外来企业高看一眼、厚爱一层、多帮一把，让外商乐于来廊坊投资，安于在廊坊兴业。营造善假于物、巧借东风的开放文化。要做足“借”字文章，是积极引进战略合作伙伴、高端人才团队，广取他山之石，实现借船出海、借梯上楼；要下足“攀”的功夫，是善于和行业实力强的合作，敢于和企业规模大的“联姻”，善于与巨人同行、与名人为伍，靠大求大、联强成强。在“借”和“攀”中，要明白“舍得”，弄清小舍小得、大舍大得、先舍后得、不舍不得的道理，开阔“今日借君一滴水明日还君一桶油”的胸怀；还要坚守前人栽树后人乘凉，不能吃祖宗饭断子孙路，注重追求更大更可持续的发展。

(五)更加推进共享，着力提高人民群众安居乐业的幸福感和获得感。

一是促进创业就业。加快市级示范性众创空间和各类创业孵化基地建设，积极搭建创业就业服务平台。落实好创业税费减免、社会保险补贴、创业担保贷款等扶持政策。开展就业困难人员帮扶，对全市1700名以上就业困难人员，建立实名制台账，实现“一对一”帮扶；做好高校毕业生、农村转移劳动力就业工作，高校毕业生就业率保持在90%以上，转移农村劳动力2.8万人。做好援企稳岗工作，及时发放稳岗补贴资金，帮助企业稳定就业。全市新增城镇就业岗位5.35万个，城镇登记失业率控制在4%以内，城镇零就业家庭保持动态清零。

二是全力做好民生保障工作。要从最弱势、最困难、最需要关怀的群众做起，积极对标京津，加大投入力度，努力提高民生保障水平。城乡低保标准由每人每月532元和354元均提高到600元，标准全省最高，率先实现城乡一体化，提前三年达到省2020年目标；农村集中供养、分散供养特困人员和城市“三无”人员基本生活补助由每人每年5750元、5000元、6384元均提高到11520元，标准全省最高；加大贫困生救助力度，为低保家庭高中生发放学费住宿费每人每年1200元、生活补助每人每年2000元，标准全省最高，达到京津水平；调整困难残疾人生活补助标准，由每人每月55元提高到255元，标准全省最高；提高孤儿救助标准，机构和散居供养标准分别由每人每月1150元、700元均提高到1440元，标准全省最高，达到京津水平；城乡居民基本养老保险基础养老金人均提高到123元，标准全省最高。

三是办好人民满意的教育。要增建校所。全市新建初中9所、小学31所、公办幼儿园22所。其中，市主城区投资8.24亿元，续建高中1所、初中2所，新建续建小学9所、公办幼儿园6所。市教育局加快廊坊一中迁建工程，年内完成四栋教学楼、两栋宿舍楼主体工程，确保2018年秋季投入使用，续建管道二小、新中家园幼儿园、瑞河兰乔小学和幼儿园；安次区续建龙河高新区第一小学、银河学校、安次三幼和第一实验小学扩建工程；广阳区新建市二十六小和二十七小，续建广阳四幼、五幼和市十六中；廊坊开发区新建开发区八小、九小、十二小和二幼。要提质攻坚。完善住宅区教育设施配建机制，出台新建住宅区配套中小学幼儿园建设管理办法，建设项目必须与教育设施同步设计、同步建设，并与一期同步验收、同步交付使用。市主城区启动教育扩容提质攻坚行动计划，三年投资35.8亿元，新增学位5.9万个，基本解决主城区“大班额、入园难”问题。要优化布局。引导县域内分散办学的农村小学适度向中心村和乡(镇)政府所在地集中，推动初中向重点镇集中、高中向县城集中；支持大厂县教育综合改革试验区建设；市主城区新建中小学重点向资源比较匮乏的北部新区和龙河园区倾斜。要配足师资。用足用好统一城乡教职工编制标准、统筹调配政策和规模适当的教师补充机制，逐步补足教师缺口，今年全市补充教师3215人。要打造名校。探索建立高端人才引进机制，在全国范围内遴选一批特级教师、省级以上优秀教师、骨干教师、学科带头人，充实我市教师队伍，带动提升学校管理水平和教育理念创新。支持廊坊一中打造省内一流高中。要促进均衡。投资1.88亿元改善义务教育薄弱学校252所，努力缩小校际差距。创建标准化学校120所，标准化学校比例达到80%。实施中职学校质量提升工程，支持东方大学城建设职教园区。要保障校安。重点开展“黑幼儿园、黑小餐桌、黑校车”专项整治，让可爱的孩子们有一个和谐平安欢乐健康的校园环境，让所有的父母都放心舒心。

四是全面推进健康廊坊建设。健全完善医疗服务体系。加快市中心医院重新选址和建设，抓紧启动市人民医院、市中医院改扩建工程；加强与北京医疗卫生资源的有效对接，积极推进和北京世纪坛、首都儿研所等医院的深度合作，填补我市儿科、肿瘤、精神卫生等专科空白；积极落实上级政策，尽快实现在京津冀区域异地就医住院费用直接结算，让廊坊人民更便捷地享受到北京优质医疗资源。深化医药卫生体制改革。全面启动主城区13家公立医院综合改革，药价平均降低15%；完善分级诊疗模式，建设城市医联体、县域医共体、京津专科联盟三级诊疗平台，开展群众满意的乡镇卫生院创建和社区卫生服务提升工程；实施家庭医生签约服务，签约覆盖率达到30%以上，努力实现小病不出村、大病不出县。完善智慧卫生体系。依托创建国家医疗大数据中心，推进廊坊区域人口健康云平台建设，实现全市常住人口居民健康卡全覆盖和区域就医“一卡通”。深入实施全面两孩政策，加强生育医疗保健服务。积极开展全民健身运动。加快推进市体育场改造提升和市滑冰馆建设，城乡健身设施覆盖面分别达到95%和80%以上；举办好永定河国际马拉松赛、全国风筝锦标赛等系列赛事活动，带动增强健身意识、开展全民健身运动，让广大群众拥有健康的体魄。

五是着力改善农村生产生活条件。大力提升农村基础设施。新改建农村公路300公里以上，路网密度保持全省第一；投资10亿元实施农村电力配网建设，提高农村供电可靠性，为“电代煤”工程提供电力保障。扎实推进美丽乡村建设。继续开展民居改造、街道硬化等“十二个专项行动”，重点推进香河30个村街连片打造省级重点片区，启动建设9个市级片区，加快建设218个省级重点村和161个市级重点村，打造一批记得住乡愁的“高颜值”美丽乡村。推进农村垃圾治理。在全省率先实现农村垃圾处理PPP模式全覆盖，所有农村垃圾全部推行无害化处理，为“煤替代”工程提供安全保障。

六是发展更加惠民的文化事业。巩固提升国家公共文化服务体系示范区创建成果，持续实施“一十百千万”文化惠民工程，组织开展百场特色品牌文化活动，建设提升2000个以上基层综合文化服务中心，推动“十大文化体验小镇”建设。健全公共文化设施，加快推进市县两级图书馆达标建设，力争全部达到国家三级馆以上标准；规划建设市级非物质文化遗产展示馆和美术馆。办好第27届全国图书交易博览会、中国图书馆年会等文化活动，积极创建国家文化消费试点城市，不断繁荣文化事业，增加文化供给，满足人民群众日益增长的精神文化需求。

七是着力提升群众安全感。深化平安廊坊建设，强化社会治安综合治理，推进公共安全视频监控“雪亮工程”，严厉打击“两抢一盗”等各类违法犯罪，积极构建“京畿平安示范区”。深入开展安全生产、食药安全专项整治，坚决遏制各类重大事故发生，切实保护好人民群众的身体健康和生命财产安全。

推进全国文明城市创建，促进市民整体素质、城市管理水平、社会文明程度全面提升。加强国防动员和国防后备力量建设，做好各项拥军工作。充分发挥工会、共青团、妇联等人民团体桥梁纽带作用，做好新闻出版广电、民族宗教、外事侨务、人民防空、气象地震、防灾减灾、史志档案、援疆援藏等工作。

全面加强政府自身建设

关于加强政府自身建设，陈平说，打铁还需自身硬，过河需要桥和船。为人民服务不是一句空话，不是一个口号，需要行动，需要落实。政府工作人员必须有党性原则、有为民情怀、有法治观念、有觉悟担当、有良好形象。

牢固坚持立党为公，始终保持对党绝对忠诚。坚持深入学习贯彻习近平总书记系列重要讲话精神，以马克思主义理论中国化的最新成果武装头脑，不断强化“四个意识”特别是核心意识和看齐意识。坚持以党的旗帜为旗帜、以党的意志为意志、以党的使命为使命。坚持以治国理政新思想新理念新要求，指导工作、推动工作、落实工作，坚决在思想上、政治上、行动上与以习近平同志为核心的党中央保持高度一致。

牢固坚持执政为民，始终坚持全心全意为人民服务。坚持以人民为中心的原则立场，带着责任、感情、追求，干事、创业、为民，想群众之所想，急群众之所急。坚持脚踏实地、真心为民，反对做表面文章、玩形式主义，力戒急功近利、搞一锤子买卖。凡是涉及百姓关心的民生事情，都要盯住盯紧，马上就办、办就办好；凡是对人民作出的承诺，都要言必行、行必果、果必真。切实把人民群众所盼的，变成政府真干的；切实把政府所干的，变成人民群众真盼的。切实以广大群众最根本利益为最大利益，以广大群众的最根本追求为最大追求。切实把为民情怀镌刻在普通群众的记忆中、心头上，把政声民意回荡在人去后、闲谈中。

牢固坚持依法行政，始终确保权力规范阳光运行。弘扬法治精神，驰而不息建设法治政府。坚持依法全面履行政府职能，做到法无授权不可为，法定职责必须为。推进依法行政制度建设，完善行政决策公众参与、专家论证、风险评估、合法性审查、集体讨论等制度。健全行政权力监督制约机制，自觉接受人大法律监督、政协民主监督、社会和舆论监督，学会在约束中工作，习惯在监督下干事。

牢固坚持实干实政，始终保持干事创业过硬作风。一定把坚强的党性落实到过硬的作风上，高调叫响要想过得去必须过得硬、只有过得硬才能过得去。一定把坚强的党性落实到从严的作风上，强力深化政府系统机关作风整顿，坚决提高行政效能，严厉治理不作为、乱作为、假作为。一定把坚强的党性落实到真抓实干的作风上，坚持夙兴夜寐、激情工作，事争一流、唯旗是夺，追求卓越、精益求精。一定把坚强的党性落实到深入实际的作风上，做到一竿子插到底，真实体察民意，全面了解实情，掌握工作主动权。一定把坚强的党性落实到敢于担当的作风上，始终有那么一股勇往直前的精气神，不回避矛盾，不躲闪困难，顶起自己该顶的那片天。

牢固坚持廉洁持政，始终注重营造风清气正政治生态。严格落实全面从严治党要求，坚决把纪律和规矩挺在前面，认真执行党内政治生活准则和党内监督条例，推动政府系统全面从严治政不断深化。严格落实中央“八项规定”精神，坚决防止“四风”反弹回潮。认真落实“两个责任”“一岗双责”，严格执行审计、监察、财政等各项制度，以“零容忍”的态度坚决查处违规、违纪、违法行为，努力建设一支政治上靠得住、工作上有本事、作风上过得硬、人民群众信得过的公务员队伍。
